# The longitudinal relationship between leisure activities and depressive symptoms among older Chinese adults: an autoregressive cross-lagged analysis approach

**DOI:** 10.1186/s12889-024-18293-4

**Published:** 2024-03-12

**Authors:** Juanjuan Wang

**Affiliations:** Department of Sociology, College of Political Science and Law, Hubei University of Arts and Science, Xiangyang, China

**Keywords:** Leisure activity, Depression, Older adults, Longitudinal relationship, Autoregressive cross-lagged model

## Abstract

**Background:**

Existing studies have shown a correlation between leisure activities and depressive symptoms in older adults, but the direction of the longitudinal relationship is inconsistent. This study used an autoregressive cross-lagged model to examine the longitudinal relationship between leisure activity participation and geriatric depression.

**Methods:**

A total of 7,138 participants aged 60 years or older from the 2nd to the 4th wave of the China Health and Retirement Longitudinal Study (CHARLS) were analysed.

**Results:**

First, present depressive symptoms were significantly associated with future depressive symptoms (*β*_*2013-2015*_ = .893, *p* < .001; *β*_*2015-2018*_ = .946, *p* < .001), and the same rule applied to leisure activities (*β*_*2013-2015*_ = .402, *p* < .001; *β*_*2015-2018*_ = .404, *p* < .001). Second, current depressive symptoms negatively predicted future leisure activities (*β*_*2013-2015*_ = –.071, *p* < .001; *β*_*2015-2018*_ = –.085, p < .001), but the inverse relationship was not statistically significant (*β*_*2013-2015*_ = –.003, *p* > .05; *β*_*2015-2018*_ = –.003, *p* > .05).

**Conclusion:**

These findings underscore the importance of interventions targeting depressive symptoms to potentially enhance engagement in leisure activities among older adults. The results contribute to the understanding of the complex dynamics between mental health and lifestyle choices in older populations, highlighting the potential of proactive mental health interventions to improve overall well-being.

**Supplementary Information:**

The online version contains supplementary material available at 10.1186/s12889-024-18293-4.

## Introduction

In China, people aged 60 and above account for 18.70% of the country's total population [1]. Studies have shown that the prevalence of depressive symptoms among middle-aged and older adults in China ranges from 20 to 27% [2]. Depressive symptoms can exacerbate symptoms of physical illness in older adults, which are often considered a result of natural aging [3], and both older adults and professionals frequently ignore these symptoms [4–7]. However, depression is a major cause of disability, suicide, and death [8–10]. Active lifestyles, particularly participation in leisure activities, play important roles in combating depression [11–14]. Conversely, individuals with depressive moods tend to engage less frequently in leisure activities [15, 16]. Although there is a strong correlation between leisure activities and depression, the direction of causality is not consistent. Service interventions and their effects may vary depending on the causal relationship between these variables [17, 18]. Therefore, there is a need to research further the longitudinal relationship between participation in leisure activities and depressive symptoms among older adults.

### Leisure activities and depressive symptoms

"Leisure activities" refer to voluntary, enjoyable, part-time activities, often described as "the principal driving force underpinning the human desire to render life meaningful… or to give it a sense of passion, pleasure, and purpose" [16, 19]. According to previous research, the protective effect of leisure activities on depressive symptoms arises from several mechanisms: First, engaging in leisure activities can evoke intrinsic positive feelings such as motivation, enjoyment, and freedom, enabling individuals to experience a range of positive emotions like happiness and pleasure [20–23]. For example, older adults may feel "useful" by participating in activities that help others and sharing their experiences. These positive emotions can help mitigate the negative emotions associated with stressful life events. Second, leisure activities, especially social interactions, provide opportunities to socialize with acquaintances, relatives, or friends, which are crucial for fostering social relationships and building informal social support networks [24, 25]. Third, exercise-based activities can improve physical functioning and enhance positive feelings and emotions in elderly individuals [26, 27]. They also provide a sense of control and enhance emotional regulation in older adults [28]. Numerous empirical studies have confirmed the protective effect of leisure activities on depressive symptoms. For instance, a cross-sectional study using data from the Michigan Cognitive Aging Project showed that lower participation in leisure activities is negatively associated with depressive symptoms [29]. A prospective cohort study also indicated that leisure-time physical activity predicted depressive symptoms 26 years later [30].

However, it is essential to note that one of the main paradigms of leisure theory is the experience of positive emotions and pleasure through participation in leisure activities [31]. However, Beck's cognitive triad theory of depression emphasizes that depressed people usually show more negative cognitions and feelings of hopelessness and have "no interest in doing anything" [32]. Lack of happiness is one of the main features of depressive symptoms, which can undermine one's ability to achieve pleasure and prevent people from gaining meaning in their lives through leisure activities [15, 31]. This means that instead of experiencing pleasure, people who experience depression may experience increased anxiety, anger, and other negative emotions during leisure activity participation [15, 33]. Over time, the more depressed they feel, the less they can benefit from leisure activities and the less frequently they participate [14, 25]. For example, an empirical study in the United States showed that depressive symptoms decreased participation in club- and hobby-based leisure activities among middle-aged and older adults [6]. In addition, a prospective community-based longitudinal study showed that among 65-year-olds in Northern Manhattan, when individuals reported higher levels of depressive symptoms, their leisure activities also decreased [34].

The theoretical and empirical research discussed above demonstrates a strong link between engaging in leisure activities and depression. Much of the research on the longitudinal relationship between leisure activity and depressive symptoms has come from developed regions [22, 35, 36], and the findings are inconsistent. For example, Son and Sung [37] used dynamic panel-data structural equation models and showed that leisure activities negatively predicted depression, but the opposite causal relationship was not statistically significant. However, studies also indicate no evidence that leisure activities predict depressive symptoms [38, 39]. Depending on the sample selection, individual leisure activity participation and mental health ratings may vary [21, 40]. This indicates that not everyone participates in leisure activities to the same extent or frequency, and personal interests, physical health, social environment, and mental health status may influence such variations. Moreover, the impact of these activities on mental health can also vary significantly depending on individual psychological conditions and life circumstances. Particularly in China, the rate of participation in leisure activities among older adults is lower than in developed regions [41]. Additionally, there is a sense of shame associated with discussing mental health issues in traditional Chinese culture, which may further influence individuals' participation in leisure activities and their assessment of mental health [42]. In this context, it is necessary to enrich research on the longitudinal relationship between leisure activities and depressive symptoms among older Chinese adults.

### The present study

Implementing an intervention often requires time before the effects are seen [18]. The time lag is one of the necessary conditions for measuring whether two variables are causally related [17, 18]. Autoregressive cross-lagged models (ARCL) can examine the predictive impact of a variable at time t on another variable at time t + 1 by setting stability coefficients [18, 43, 44], which are often used to measure the reciprocal relationship between two variables [45]. Therefore, this study aims to examine the longitudinal relationship between depressive symptoms and leisure activity participation among older adults using the ARCL based on three waves of data. Two research hypotheses were proposed along with existing theoretical and empirical studies: (1) current depressive symptoms can predict future leisure activity participation, and (2) current leisure activities can predict future depressive symptoms.

## Method

### Data and samples

**Fig. 1:**
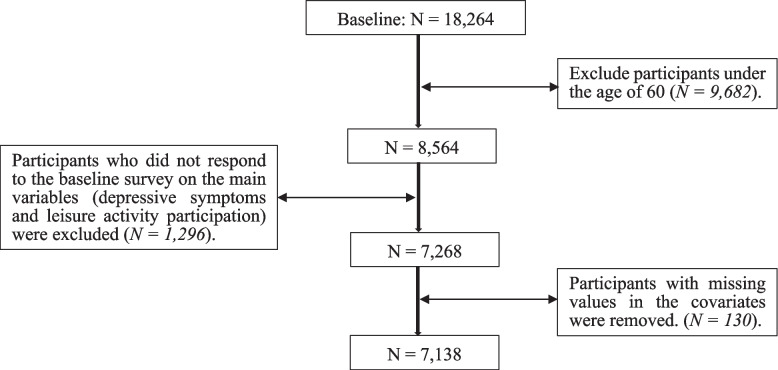
Participant sampling procedure

This study used data from the China Health and Retirement Longitudinal Study (CHARLS), which covered 150 randomly selected counties and districts, 450 village residences, more than 10,000 households, and approximately 18,000 individuals aged 45 and older across the country [46]. The first wave of the CHARLS national sample was conducted in 2011, and the second, third, and fourth waves of the survey were successfully conducted in 2013, 2015, and 2018. For this study, the baseline was 2013 because 2,776 new respondents were added in 2013. The CHARLS interviewed 18,264, 20,284, and 17,970 individuals in 2013, 2015, and 2018, respectively. A sample of older adults aged 60 years and older at baseline and without missing values for depressive symptoms and leisure activities in 2013 was included in this study, which means that the 7,138 samples used for analysis in 2013 are free of missing values (The sample selection process is shown in Fig. 1). By ID matching, 6,385 and 5,746 samples were used for analysis in 2015 and 2018, respectively.

### Measures

#### Depressive symptoms

Depressive symptoms, characterized by a difficult mood or negative psychological state, can significantly impact daily activities such as sleep, judgment, and interpersonal relationships [47]. To measure these symptoms, Radloff (1977) developed the Center for Epidemiologic Studies-Depression Scale (CES-D), a widely used tool for depression screening [48]. In the CHARLS study, depression was assessed using 10 items from the CES-D, with 8 negative items (e.g., 'I was depressed') and 2 positive items (e.g., 'I was happy'). The positive items were reverse-scored, all summed to indicate depressive symptoms severity.

This study conceptualized depressive symptoms as a latent variable based on the division of positive and negative moods. The CES-D-10 scale, a validated screening tool, assesses depressive symptoms by measuring both positive and negative emotional states. Cut-off values for depressive symptoms were set following guidelines by Andresen et al. [48] and subsequent research [49, 50], aiding in distinguishing between normal mood states and potential depressive symptoms. This research model uses latent variables to show complicated emotional or psychological states. These variables fix measurement errors in observed variables, which leads to more accurate estimates of depressive symptoms [51]. The high internal consistency of the CES-D is evidenced by Cronbach's alpha values of 0.760, 0.795, and 0.802 in 2013, 2015, and 2018, respectively.

#### Leisure activity

Leisure activities are commonly defined as voluntary, non-work activities for enjoyment. There has yet to be a consensus among scholars regarding measuring leisure activities. Building upon previous research [6, 14, 16, 33], this study employed a 6-item measure to assess leisure activities. The six items are: "Played Ma-jong, played chess, played cards, or went to a community club"; "Interacted with friends"; "went to a sport, social, or other kind of club"; "Took part in a community-related organization"; "Done voluntary or charity work"; and "Attended an educational or training course." In CHARLS, individuals were asked whether they participated in the activity (yes = 1, no = 0), and those who answered "yes" were further asked about the frequency of their participation. The answers were "1 = Not regularly", "2 = Almost every week", and "3 = Almost daily". The total score for each item was added up and ranged from 0 to 18, with higher scores indicating greater participation in leisure activities. The Cronbach's alpha values of the leisure activities in 2013, 2015, and 2018 were 0. 929, 0. 919, and 0. 952, respectively, suggesting the scale's high internal consistency.

#### Control variables

This study includes several control variables based on existing research: basic demographic factors (age, sex, marital status, education level), lifestyle choices (smoking and drinking alcohol), health status (self-rated health and chronic diseases), and economic conditions (per capita household income). The selection of these variables is informed by their established correlation with depressive symptoms and lifestyle activities in previous studies. For example, research indicates a higher prevalence of depression among older women than men [19]. Factors such as age, marital status, and educational level are known to influence social support networks and coping mechanisms, impacting mental health. Older individuals with less than elementary education or who are single have been shown to have higher depression scores [5]. Physical activity participation is noted to reduce the risk of depression, particularly in those aged 60–69 [19]. Additionally, economic status and health-related factors significantly affect the incidence of depressive symptoms and the frequency of participation in leisure activities [6]. Lifestyle choices like smoking and drinking are closely associated with an individual's physical and psychological well-being [52]. By inquiring about participants' smoking and alcohol consumption in the past year, the study aims to contain the potential impact of these habits on health and mental state.

As for the measurement of the control variables, age is treated as a continuous variable. In CHARLS, gender is reported as male (0) or female (1), with no option for non-binary genders. Marital status is categorized as married (including married and long-term cohabitation) = 1, and other marital statuses (including single, divorced, and widowed) = 0. Education level is classified as elementary education or below = 0, and secondary education or higher = 1. Smoking and drinking alcohol consumption were assessed by asking participants if they had smoked cigarettes or consumed alcohol in the past year, with responses coded as 1 for yes and 0 for no. Self-rated health was measured on a 5-point Likert scale, ranging from 1 (very poor) to 5 (very good). In CHARLS, participants are asked whether they have been diagnosed with any of 14 common chronic diseases, including hypertension and diabetes. The presence of each chronic disease is recorded individually, and the total number of chronic diseases recorded is summed up. Based on this total number, participants are categorized into the following groups: 'No Chronic Disease' (0 chronic diseases), '1 Chronic Disease', '2 Chronic Diseases', and '3 or More Chronic Diseases'. This categorization allows us to assess the impact of the chronic disease burden on the study results while providing a simplified method for describing the health status of the participants.

### Statistical analysis

This study analyzed the interaction causality between leisure activity participation and depression using an autoregressive cross-lagged model (ARCL), which is a structural equation model specifically designed for panel data that reveals the interaction of different variables over time [53]. ARCL has two main components: autoregressive and cross-lagged models [18]. Autoregressive models can observe the predictive impact of engaging in activity at time t on itself at time t + 1, i.e., analyze the stability of the measured variable across time [54, 55]. Cross-lagged models can assess the effect of leisure activity participation at time t on depression at time t + 1, and vice versa. The cross-lagged coefficient is an essential parameter for proving mutual causation [55], and it shows how two variables interact over time [54]. The equation for the ARCL is as follows:

1 $$y_{it}=\alpha y_t+\rho y_ty_{t-1}y_i,_{t-1}+\rho y_tw_{t-1}w_i,_{t-1}+\in y_{it},$$

2 $$w_{it}=\alpha w_t+\rho w_ty_{t-1}y_i,_{t-1}+\rho w_tw_{t-1}w_i,_{t-1}+\in w_{it}$$

In this equation, y is the latent variable of depression, w is the observable variable of leisure activity participation, and *α*_*yt*_ and *α*_*wt*_ are the (fixed) intercepts for the equation for time *t* [53]. ρ_ytyt-1_ and ρ_wtwt-1_ are autoregressive coefficients of depressive symptoms and leisure activity, respectively, and ρ_ytwt-1_ and ρ_wt yt-1_ are cross-lagged coefficients of variables, respectively. ∈ _*yit*_ and ∈_*wit*_ are the residuals for individual i at time t. These residuals are assumed to be cross-sectionally correlated in the ARCL model.

In this study, the best model was selected to verify the research hypothesis by comparing the fit of seven models. M1 is the base model without any restrictions. It allows us to observe the natural progression of each variable without constraints. M2 is a fixed factor loading on the depressive symptom variable across time to test the assumption that how depressive symptoms manifest remains consistent. M3: Restricted depressive symptoms autoregressive coefficients are equal across time to examine the stability of these symptoms over time. M4: Applied the same constraint as M3 for leisure activity participation to assess its temporal stability. M5: Restricted cross-lagged coefficients from depressive symptoms to leisure activity, suggesting that the influence of depressive symptoms on subsequent leisure activity does not change over time. M6: Similar to M5, but examined the influence of leisure activity on subsequent depressive symptoms. M7: Equalized the residuals between depressive symptoms and leisure activity participation across time, exploring the assumption that the unexplained variance in the relationship between the two variables is consistent. It should be noted that the latter models are all constraints added to the previous model.

All basic statistics in this study were performed using SPSS.26. The analysis of the longitudinal relationship was performed using AMOS.23. χ^2^(chi-square), RMSEA (root mean square error of approximation), SRMR (standardized root mean square), CFI (comparative fit index), and TLI (Tucker-Lewis’s index) were used to rate the model fit. χ^2^: it's a fundamental indicator of the discrepancy between the observed and estimated covariance matrices, offering an initial assessment of fit. RMSEA helps us assess how well the model, with unknown but optimally chosen parameter estimates, would fit the population's covariance matrix. SRMR: This index is an absolute measure of fit and represents the standard deviation of the residuals. It’s a measure of the average discrepancy between the observed correlations and the model's predicted correlations. CFI and TLI: Both are incremental fit indices that compare the specified model to a baseline model, typically a null model with no relations among variables. Generally, researchers compare the fit of models utilizing chi-square difference testing, which is sensitive to sample size [53]. CFI and RMSEA are less sensitive to estimation methods and sample size, especially with larger samples, making them reliable indicators for evaluating model fit in SEM [56]. Therefore, this study will compare models with indicators such as CFI and RMSEA. When the difference in CFI (ΔCFI) between two adjacent models does not exceed 0.01 and the difference in RMSEA (ΔRMSEA) does not exceed 0.015, it indicates that the model's fit did not worsen even with increasing constraints [10, 57]. When CFI and TLI are above.90, SRMR, and RMSEA are below.08, indicating a good model fit [53]. In statistical model, the analysis of whether the regression coefficients are significant is based on the p-value. A p-value less than 0.05 was considered statistically significant. This threshold indicates less than a 5% probability that the observed associations or effects occurred by chance. Findings with a p-value below 0.05 will likely have enough evidence to suggest an actual effect or association rather than just random differences in the data.

### Missing data and sensitivity analyses

Approximately 20% of participants were lost for the final interview in 2018. Missing data for key variables may stem from a variety of factors. For the variable of depressive symptoms, possible reasons include the health status of the participant, especially as individuals who may be suffering from severe depressive symptoms may have avoided participating in the survey or withdrawn during the survey. In addition, social and cultural factors may also play a role in that, in some cultures, there are still barriers to discussing mental health issues. For example, in traditional Chinese culture, acknowledging mental health problems may be seen as a personal weakness, and therefore, individuals will avoid discussing them [58]. In addition, although mental health services are gradually developing in China, awareness of mental health problems and access to mental health services are still insufficient [59]. As for the variable of leisure activity participation, the missing variable may be related to age, physical ability, or opportunities for social participation.

This study conducted independent samples t-tests on the baseline and full samples. It was found that there were significant differences between the two samples on other demographic variables, except for non-significant differences in per capita household income and alcohol consumption. This suggests that the sample differences limit the generalization of the findings to the broader population and imply that the absence of critical variables may not be random. In order to ensure the robustness of our study findings, we conducted a sensitivity analysis. This step is crucial for ensuring our results are not influenced by assumptions or missing data [60]. Initially, we performed an analysis utilizing a subset of our data that contained no missing values from 2013 to 2018 to confirm the consistency of our results with a complete case dataset. Subsequently, we expanded our dataset to include observations from 2011, increasing the temporal scope of our analysis. This extension aimed to assess the persistence of observed relationships over a longer duration and under varying conditions. The sensitivity analysis procedures were carried out by systematically re-estimating our model parameters using these alternative datasets, thus allowing us to evaluate the stability of our findings across different data scenarios and enhance confidence in our conclusions [60, 61].

## Results

### Descriptive statistics

Table 1 displays the essential characteristics of the 7,138 participants in 2013 (mean age = 68; SD = 6.57). Male and female were roughly equal in number, with the majority being married (80.0%), having an educational level of elementary school or below (80.0%), not smoking (58.0%), and not drinking alcohol (68%). Approximately half (50.9%) of the respondents rated their health as fair. The mean number of chronic conditions was 1.4. Additionally, the average per capita annual household income value was 2812.2 RMB. The mean score for depressive symptoms among older adults increased from 8.17 in 2013 to 8.85 in 2018. In contrast, the mean score for leisure activities decreased from 1.59 to 1.20. Table 2 demonstrates the means and longitudinal correlation analysis between depressive symptoms and leisure activities.

**Table 1:** Basic characteristics of the participants (*N* = 7,138 in baseline)

Variable / Categories	N (%)
**Age**	
60–69 years	4,616 (64.7)
70–79 years	2,046(28.6)
80 +	476(6.7)
**Sex**	
Male	3,592(50.3)
Female	3,546(49.7)
**Marital status**	
Married	5,708 (80.0)
Other marital status	1,430 (20.0)
**Education level**	
Elementary education and below	5,728 (80.2)
Secondary education and higher	1,410 (19.8)
**Smoking**	
Yes	2,999(42.0)
No	4,139(58.0)
**Drink alcohol**	
Yes	2,281(32.0)
No	4,857(68.0)
**Self-rated health**	
Very poor	412(5.8)
Poor	1,607(22.5)
Fair	3,632(50.9)
Good	961(13.5)
Very good	526(7.4)
**Chronic diseases**	Mean = 1.4 (SD = 1.11)

Per capita annual household income (mean = 2812.2; SD = 6356; Min = 459, Max = 136,900 RMB)

**Table 2:** Means and longitudinal correlations of the main variables

Variable	1	2	3	4	5	6
1.Depression _T1_	1					
2.Depression _T2_	.528^**^	1				
3.Depression _T3_	.484^**^	.522^**^	1			
4.Leisure activities _T1_	-.150^**^	-.110^**^	-.098^**^	1		
5.Leisure activities _T2_	-.110^**^	-.129^**^	-.105^**^	.423^**^	1	
6.Leisure activities _T3_	-.080^**^	-.112^**^	-.096^**^	.377^**^	.429^**^	1
Mean	8.17	8.56	8.85	1.59	1.36	1.20
SD	5.841	6.567	6.584	1.943	1.835	1.721
Kurtosis	.601	.147	-.082	1.659	1.895	2.846
Skewness	.933	.865	.753	1.284	1.419	1.599

^**^p < 0.01(2-tailed)

### Longitudinal relationship analysis: ARCL

**Fig. 2:**
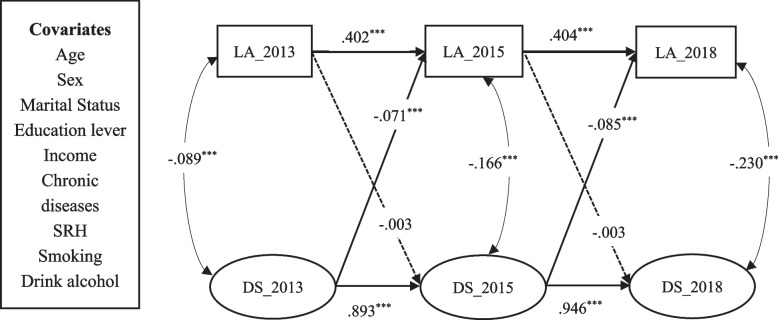
Final structural equation diagram. Note: *** *p*<.001, LA = leisure activities, DS = depressive symptoms, Income = per capita household income, SRH = self-rated health

This study examined bidirectional causality through the order of measurement invariance (M2), structural invariance (M2–6), and residual invariance (M7). Table 3 shows a comparison of the models for ARCL, the △CFI did not exceed 0.01, and △RMSEA did not exceed 0.015, which means that the model's fit did not worsen even with increasing constraints [10, 56, 57]. In this context, the model with the most restrictive parameters and the simplest Model 7 is the optimal model to explain the longitudinal relationship. The final structural diagram is shown in Fig. 2. It is important to note that all models are derived after including control variables.

**Table 3:** Model comparison of ARCL

**Model**	**χ**^**2**^	**df**	**CFI**	**TLI**	**RMSEA** [90% CI]	**△χ**^**2**^	**△df**	**△CFI**
1	564.527	76	.967	.925	.031[.028 .033]	-	-	-
2	569.175	78	.967	.927	.030[.028 .033]	4.649	2	.000
3	569.289	79	.967	.928	.030[.028 .033]	.114	1	.000
4	594.658	80	.965	.925	.031[.029 .033]	35.369	1	-.002
5	594.829	81	.965	.926	.031[.028 .033]	.171	1	.000
6	594.843	82	.965	.927	.030[.028 .033]	.014	1	.000
**7**	**642.457**	**84**	**.962**	**.923**	**.031[.029 .034]**	**47.628**	**2**	**-.003**

The table presents the fit of the data after adding the control variables

Table 4 illustrates the path coefficients of the final model. First, there are significant autoregressive coefficients for leisure activity (depressive symptoms), which means that leisure activity at time t–1 significantly predicts leisure activity at time t (*β*_*2013-2015*_ = 0.402, *p* < 0.001; *β*_*2015-2018*_ = 0.404, *p* < 0.001), as does depressive symptoms (*β*_*2013-2015*_ = 0.893, *p* < 0.001; *β*_*2015-2018*_ = 0.946, *p* < 0.001). Second, depressive symptoms at the previous time negatively predicted leisure activity participation at the next time point in terms of the cross-lagging coefficient (*β*_*2013-2015*_ = –0.072, *p* < 0.001, *β*_*2015-2018*_ = –0.083, *p* < 0.001). However, leisure activity participation at the previous time did not significantly predict future depressive symptoms (*β*_*2013-2015*_ = –0.003, *p* > 0.05; *β*_*2015-2018*_ = –0.003, *p* > 0.05). Third, errors in leisure activity and depressive symptoms were negatively correlated at the cross-sectional level (e.g., *β*_*2013*_ = –0.097, *p* < 0.001, in wave 1).

**Table 4:** Path coefficients of the final model

Paths			B	*β*	*SE*	C.R
Autoregressive path						
L_1	→	L_2	.381	.402^***^	.009	40.946
L_2	→	L_3	.381	.404^***^	.009	40.946
D_1	→	D_2	1.013	.893^***^	.021	47.237
D_2	→	D_3	1.013	.946^***^	.021	47.237
Cross-lagged path						
L_1	→	D_2	-.005	-.003	.024	-.213
L_2	→	D_3	-.005	-.003	.024	-.213
D_1	→	L_2	-.043	-.071^***^	.007	-5.990
D_2	→	L_3	-.043	-.085^***^	.007	-5.990
Covariance						
D_1		L_1	-.412	-.089^***^	.064	-6.397
D_2		L_2	-.412	-.166^***^	.064	-6.397
D_3		L_3	-.412	-.230^***^	.064	-6.397

^***^ *p* < .001, *L *leisure activities, *D *Depressive symptoms, *B *Unstandardized coefficients, *β *Standardized coefficient, *SE *Standard error, *C.R. *Critical ratio, Covariance is the estimated correlation between leisure activities' error and depressive symptoms' error

To ensure the robustness of the findings, we performed a sensitivity analysis. The preliminary analysis used a subset of data with no missing values from 2013 to 2018 to confirm the consistency of the results. We subsequently extended the data to 2011 to assess the persistence of the observed relationships over longer time horizons. The results stay the same across a range of data scenarios. We show that our results are robust by re-estimating the model parameters using these different data sets. Results from the sensitivity analysis are presented in the appendix (Supplemental Table S1.1, Table S1.2, Table S2.1, Table S2.2).

## Discussion

This study examined the longitudinal causal relationship between leisure activity participation and depressive symptoms in an older population using the ARCL approach. The findings showed that current depressive symptoms negatively predicted future leisure activity participation, but the reverse causality was not statistically significant.

The depression score can predict subsequent participation in leisure activities, supporting previous research [62, 63]. It emphasizes that low levels of engagement in leisure activities are long-term consequences of higher depressive symptoms rather than temporary ones. Measures to reduce or prevent depressive symptoms may lower the risk of long-term nonparticipation in leisure activities. Although in our common sense, depressive symptoms are often thought of as a result, not a cause. In actuality, Beck's Cognitive Theory of Depression [32] can explain the results of this study. Specifically, Beck's theory emphasizes the role of negative thought patterns in the development and maintenance of depressive symptoms. Older adults with depressive symptoms may develop negative perceptions of themselves and their abilities. They may believe that they are no longer capable of or worthy of leisure activities. Second, the feelings of hopelessness in depressive symptoms may lead to a lack of interest in activities that once brought pleasure, including leisure activities. Third, individuals with depressive symptoms may focus more on their limitations than on their abilities, leading to a lower likelihood of initiating or persisting in leisure activities due to perceived incompetence or fear of failure. Over time, negative perceptions of self and the future may lead older adults to disengage from leisure activities. Additionally, the first result of this study can be explained by Activity Theory [64], which claims that active participation in social and recreational activities is critical to happiness and well-being, especially in old age. Retirement and aging often result in the loss of social roles (e.g., work roles), which leisure activities can partially mitigate. Depressive symptoms can prevent older adults from engaging in these compensatory activities, affecting their sense of purpose and belonging. As depressive symptoms worsen, older adults' tendency to engage in the social aspects of leisure activities diminishes, which may lead to social isolation. Nonetheless, active participation in leisure activities has been recognized as a primary source of syndromes and psychological well-being in later life [65], which implies that health providers should recognize or intervene early for depressive symptoms in older adults. In addition, in China, there is no standardized measurement tool for leisure activities, and practitioners can use the Depression Scale to assess older adults' participation in leisure activities.

Furthermore, contrary to our expectations, leisure activities cannot predict future depressive symptoms, which is inconsistent with the findings of Henning et al. [14] and Son and Sung [37]. Those studies demonstrated that leisure activities can predict future depressive symptoms. However, the results of this study support an earlier prospective study, which suggests that the risk of developing depressive symptoms is the same for nonengagement and engagement in physical activities [38]. Similarly, a meta-analysis study revealed that out of 30 studies, 25 demonstrated that physical activity can predict depressive symptoms. However, five studies were still related to samples of elderly individuals who failed to establish a link between physical activity and predicting depressive symptoms [39]. Furthermore, Henning et al. [14] attempted to analyze the bidirectional causal relationship between leisure activities and depressive symptoms using a bivariate dual change score model. However, the crucial parameters (i.e., coupling parameters) in the model were insignificant, thus failing to confirm a clear direction of causality.

There are two possible explanations. One possible explanation is the lower participation rate in leisure activities among older adults in this study. The mean values for leisure activities presented in Table 2 indicate that the older adults in this study had low levels of participation in leisure activities. For example, a study by Kim et al. [66] showed that the average level of participation in sedentary leisure activities among older adults in the U.S. was 19.1, and the average level of participation in community-based leisure activities was 14.0. The levels of participation in both types of leisure activities are much higher than those observed in the present study. Given the data limitations, a direct comparison with previous studies is beyond the scope of this research paper. However, this is a crucial avenue for future research to determine if these lower levels are unique to our sample or an emerging trend in the older adult population.

Another possible explanation (and perhaps the more important one) is the need to consider an individual's satisfaction with their level of engagement in leisure activities. Socioemotional Selectivity Theory asserts that as people age, they are more inclined to pursue emotionally relevant goals than informational or intellectual ones [67]. According to this theory, older adults may be more inclined to choose activities that provide emotional satisfaction. This means that the quantity of leisure activities is not necessarily the critical factor influencing depressive symptoms in older adults. Instead, the quality of these activities and their role in providing emotional support may be more important. Specifically, satisfaction with leisure activities is a complex construct reflecting engagement quantity and quality. It encompasses personal enjoyment, perceived value, social interaction, and a sense of accomplishment [68]. High satisfaction might enhance the protective effects of leisure activities against depressive symptoms by improving mood, providing a sense of purpose, and fostering social connections [69]. Conversely, low satisfaction or engagement in activities not aligning with an individual's interests or needs might not confer the same mental health benefits. This nuanced understanding of leisure activity satisfaction may explain the variance in its predictive power for depressive symptoms among older adults. That being said, leisure activity satisfaction may be an essential mediator between leisure activity and mental health outcomes. Unfortunately, the database used for the analysis of this study did not contain a measure of satisfaction with leisure activities. The results of this study emphasize the need for a multifaceted approach when examining the therapeutic potential of leisure activities. Healthcare providers should be sensitive to the importance of emotional responses in leisure participation and consider individual preferences and satisfaction levels when recommending leisure activities as part of mental health interventions.

In our study, there is a significant covariance between the residuals of leisure activities and depressive symptoms, which suggests a substantial, yet non-causal, association between these two variables. This finding enriches our understanding by indicating a complex interplay beyond straightforward causal relationships. Notably, this covariance highlights an indirect association, where factors like an individual's perceptions, attitudes, and the socio-emotional value of leisure activities play a crucial role. It points to the need for future research to delve into these indirect pathways, exploring how subjective factors and personal experiences of leisure activities contribute to their impact on depressive symptoms. This nuanced perspective opens up new avenues for research, focusing on the qualitative aspects of leisure activities and their psychological implications rather than solely on their frequency or intensity.

The results of this study have important implications for China's current depression symptom prevention policies and their improvement. Since 2002, China has, through measures such as the development of China's 2002–2010 Mental Health Work Plan and the National Mental Health Work Plan (2015–2020), made progress in mental health services, especially in integrating hospital and community mental health services [42, 70]. However, policies have primarily focused on the treatment of severe mental illnesses rather than the prevention of prevalent mental illnesses, such as depressive symptoms. Given the relationship between depressive symptoms and leisure activity participation in the older adult population, prevention policies should place greater emphasis on promoting older adults' participation in leisure activities as a way to reduce depressive symptoms, even though the present study did not find a nonsignificant direct predictive effect of leisure activities on future depressive symptoms. Our findings still support that Chinese policymakers should continue to explore and strengthen promotional strategies for older adults' leisure activity participation as part of a comprehensive depression prevention program. Leisure activities may have preventive effects on depressive symptoms through non-directive pathways such as improving quality of life, increasing social interactions, and enhancing personal well-being [69, 71]. Future policies should consider the needs of older people in terms of satisfaction and quality of participation in leisure activities and use this as a basis for designing and implementing relevant interventions. Encouraging older adults to participate in leisure activities that bring personal satisfaction and enhance well-being may help alleviate depressive symptoms and improve mental health [72].

Additionally, considering the impact that depressive symptoms may have on the long-term participation of older adults in leisure activities, prevention strategies should focus on early identification and intervention of depressive moods. Such identification and intervention can be conducted through community centers, primary care services, or other facilities frequented by older adults. For instance, community centers can offer regular mental health screening events, and primary care services can include mental health assessments as part of routine health check-ups. These measures can help detect potential depressive symptoms early and prevent them from worsening by providing timely support and intervention. In addition, educational activities and workshops can be organized by the community and primary health care services to raise awareness of depression among older adults and encourage them to seek help actively. Through such strategies, depressive symptoms can be effectively reduced, the quality of life of older people can be improved, and their more active participation in community life and leisure activities can be promoted.

To the best of my knowledge, our study is one of the few longitudinal studies on leisure activities and depressive symptoms in China. Moreover, the results of the two sensitivity analyses were consistent with this study's main findings, ensuring the findings' stability and validity. This analytical approach ensures that our conclusions apply to a specific subset of data and have broader applicability. Nevertheless, it is essential to consider the following limitations when interpreting the results of this study. First, the participants in this study were community-dwelling older adults, which may result in lower scores for depressive symptoms. Community-dwelling older adults may have had better health and would have had fewer depressive symptoms compared with the clinical sample, which may underestimate or overestimate the cross-lagged associations. Specifically, with this group reporting lower depressive symptoms, it would be harder for leisure activity participation to go on to influence mood changes. Therefore, we suggest that this should be considered when generalizing the findings to broader populations or settings where depressive symptoms may be more prevalent. Second, while measuring through recall allows us to capture subjective experiences over time, it also introduces the possibility of systematic error. Specifically, the variable measuring leisure activity participation was based on older adults' recollections of the past year, which might lead to overestimation or underestimation of their frequency of participation. Similarly, depressive symptoms were assessed based on recall over the past two weeks, and there is a possibility that older adults might overestimate or underestimate changes in their mood. This potential systematic bias could distort the observed relationship between the variables. Future research would benefit from utilizing more contemporaneous or objective measures of leisure activities and mood to reduce the impact of recall bias. Future research could consider using clinical data for verification. Third, although this study considered the frequency of engagement in leisure activities, it did not assess participants' satisfaction with these activities or the intensity of their involvement due to the limitations of the questionnaire. Future studies could explore incorporating these factors.

## Conclusion

The present study's analysis of the longitudinal relationship between leisure activities and depressive symptoms in community-dwelling older adults reveals a significant, yet non-causal, association between these variables. The observed covariance underscores the complex interplay beyond direct causal links, highlighting the importance of understanding leisure activities' quantitative and qualitative aspects. While depressive symptoms predict future leisure activity levels, the inverse is insignificant, suggesting the need to explore bidirectional or cyclical interaction patterns. Future research should explore how various factors interplay with these variables, including social support, physical health, and life quality. The insights gained are crucial for developing effective interventions targeting the mental health and overall well-being of older adults, emphasizing the frequency and emotional and social value of leisure activities.

### Supplementary Information

**Supplementary Material 1.**

## Data Availability

Raw data were generated at China Health and Retirement Longitudinal Study (CHARLS). Derived data supporting the findings of this study are available from the corresponding author on request.
