# Supplementary material for: The longitudinal relationship between leisure activities and depressive symptoms among older Chinese adults: an autoregressive cross-lagged analysis approach
Source: BMC Public Health. 2024 Mar 12;24:763. doi: 10.1186/s12889-024-18293-4 (PMC10929180; doi:10.1186/s12889-024-18293-4)
Supplement: Supplementary file 1 — Supplementary Material 1. [file 12889_2024_18293_MOESM1_ESM.docx]

**Appendix 1-Sensitivity analyses**

**Study 1. 2013-2018 complete sample**

Table S1.1 Model comparison of ARCL

| **Model** | **χ^2^** | **df** | **CFI** | **TLI** | **RMSEA** [90% CI] | **△χ^2^** | **△df** | **△CFI** |
| --- | --- | --- | --- | --- | --- | --- | --- | --- |
| 1 | 665.001 | 76 | .957 | .904 | .040[.037 .042] | - | - | - |
| 2 | 668.026 | 78 | .957 | .906 | .039[.036 .042] | 3.024 | 2 | .000 |
| 3 | 668.058 | 79 | .957 | .908 | .039[.036 .042] | .032 | 1 | .000 |
| 4 | 678.264 | 80 | .957 | .907 | .039[.036 .042] | 10.206 | 1 | .000 |
| 5 | 678.446 | 81 | .957 | .909 | .039[.036 .041] | .182 | 1 | .000 |
| 6 | 678.892 | 82 | .957 | .910 | .038[.036 .041] | .446 | 1 | .000 |
| **7** | **715.645** | **84** | **.954** | **.907** | **.039[.036 .042]** | **36.754** | **2** | **-.003** |

*Note:* The table presents the fit of the data after adding the control variables.

Table S1.2 Path coefficients of the final model

| **Paths** | | | **B** | ***β*** | ***SE*** | **C.R.** |
| --- | --- | --- | --- | --- | --- | --- |
| Autoregressive path | | |  |  |  |  |
| L_1 | → | L_2 | .395 | .421^***^ | .009 | 45.118 |
| L_2 | → | L_3 | .395 | .416^***^ | .009 | 45.118 |
| D_1 | → | D_2 | .987 | .904^***^ | .018 | 54.628 |
| D_2 | → | D_3 | .987 | .928^***^ | .018 | 54.628 |
| Cross-lagged path | | |  |  |  |  |
| L_1 | → | D_2 | -.002 | -.001 | .022 | -.091 |
| L_2 | → | D_3 | -.002 | -.001 | .022 | -.091 |
| D_1 | → | L_2 | -.033 | -.057^***^ | .006 | -5.192 |
| D_2 | → | L_3 | -.033 | -.066^***^ | .006 | -5.192 |
| Covariance | | |  |  |  |  |
| D_1 |  | L_1 | -.417 | -.089^***^ | .063 | -6.629 |
| D_2 |  | L_2 | -.417 | -.171^***^ | .063 | -6.629 |
| D_3 |  | L_3 | -.417 | -.195^***^ | .063 | -6.629 |

*Note*: *** p<.001, L = leisure activities, D = depressive symptoms, B = Unstandardized coefficients, *β* = Standardized coefficient, SE = standard error, C.R. = critical ratio, Covariance is the estimated correlation between leisure activities' error and depressive symptoms' error.

**Study 2. 2011-2018 sample**

Table S2.1 Model comparison of ARCL

| **Model** | **χ^2^** | **df** | **CFI** | **TLI** | **RMSEA** [90% CI] | **△χ^2^** | **△df** | **△CFI** |
| --- | --- | --- | --- | --- | --- | --- | --- | --- |
| 1 | 1252.081 | 130 | .951 | .904 | .036[.034 .038] | - | - | - |
| 2 | 1262.099 | 133 | .950 | .905 | .035[.034 .037] | 10.081 | 3 | .001 |
| 3 | 1264.620 | 135 | .950 | .907 | .035[.033 .037] | 2.521 | 2 | .002 |
| 4 | 1406.542 | 137 | .944 | .900 | .037[.035 .039] | 141.922 | 2 | -.007 |
| 5 | 1410.342 | 139 | .944 | .900 | .037[.035 .038] | 3.799 | 2 | .000 |
| 6 | 1410.794 | 141 | .944 | .900 | .036[.035 .038] | .452 | 2 | .000 |
| **7** | **1465.835** | **144** | **.942** | **.900** | **.037[.035 .039]** | **55.042** | **3** | **-.002** |

*Note:* The table presents the fit of the data after adding the control variables.

Table S2.2 Path coefficients of the final model

| **Paths** | | | **B** | ***β*** | ***SE*** | **C.R.** |
| --- | --- | --- | --- | --- | --- | --- |
| Autoregressive path | | |  |  |  |  |
| L_1 | → | L_2 | .382 | .358^***^ | .008 | 49.255 |
| L_2 | → | L_3 | .382 | .399^***^ | .008 | 49.255 |
| L_3 | → | L_4 | .382 | .404^***^ | .008 | 49.255 |
| D_1 | → | D_2 | .826 | .935^***^ | .011 | 74.707 |
| D_2 | → | D_3 | .826 | .805^***^ | .011 | 74.707 |
| D_3 | → | D_4 | .826 | .871^***^ | .011 | 74.707 |
| Cross-lagged path | | |  |  |  |  |
| L_1 | → | D_2 | -.027 | -.015 | .017 | -1.536 |
| L_2 | → | D_3 | -.027 | -.015 | .017 | -1.536 |
| L_3 | → | D_4 | -.027 | -.015 | .017 | -1.536 |
| D_1 | → | L_2 | -.059 | -.115^***^ | .005 | -11.530 |
| D_2 | → | L_3 | -.059 | -.106^***^ | .005 | -11.530 |
| D_3 | → | L_4 | -.059 | -.115^***^ | .005 | -11.530 |
| Covariance | | |  |  |  |  |
| D_1 |  | L_1 | -.368 | -.073^***^ | .049 | -7.553 |
| D_2 |  | L_2 | -.368 | -.186^***^ | .049 | -7.553 |
| D_3 |  | L_3 | -.368 | -.115^***^ | .049 | -7.553 |
| D_4 |  | L_4 | -.368 | -.156^***^ | .049 | -7.553 |

*Note*: *** p<.001, L = leisure activities, D = depressive symptoms, B = Unstandardized coefficients, *β* = Standardized coefficient, SE = standard error, C.R. = critical ratio, Covariance is the estimated correlation between leisure activities' error and depressive symptoms' error.
